# The effects of supraphysiological levels of testosterone on neural networks upstream of gonadotropin-releasing hormone neurons

**DOI:** 10.22038/ijbms.2019.36127.8605

**Published:** 2019-09

**Authors:** Mohammad Saied Salehi, Homayoun Khazali, Fariba Mahmoudi, Mahyar Janahmadi

**Affiliations:** 1 Clinical Neurology Research Center, Shiraz University of Medical Sciences, Shiraz, Iran; 2 Department of Animal Physiology, Faculty of Life Sciences and Biotechnology, Shahid Beheshti University, Tehran, Iran; 3 Faculty of Basic Sciences, University of Mohaghegh Ardabili, Ardabil, Iran; 4 Neuroscience Research Center and Department of Physiology, Faculty of Medicine, Shahid Beheshti University of Medical Sciences, Tehran, Iran

**Keywords:** GnRH, Kisspeptin, Neurokinin B, RFRP, Testosterone

## Abstract

**Objective(s):**

Several pathological conditions are associated with hyper-production of testosterone; however, its impacts are not well understood. Hence, we evaluated the effects of supraphysiological levels of testosterone on gonadotropin-releasing hormone (GnRH) system in the hypothalamus of male rats. Also, we assessed the expression of two excitatory (kisspeptin and neurokinin-B) and two inhibitory (dynorphin and RFamide-related-peptide) neuropeptides upstream of GnRH neurons as possible routes to relay androgen information.

**Materials and Methods:**

Gonadectomized (GDX) male rats received single injection of 100, 250 or 500 mg/kg testosterone undecanoate and three weeks later, posterior (PH) and anterior (AH) hypothalamus was dissected for evaluation of target genes using quantitative RT-PCR.

**Results:**

We found that GnRH mRNA in the PH was high in GDX rats and 500 mg/kg testosterone reduced GnRH level expression. Finding revealed extremely high level of Kiss1 mRNA in the PH of GDX rats. However, in GDX rats treated with different levels of testosterone, Kiss1 expression was not significantly different than control. We also found that testosterone replacement increased the Kiss1 mRNA level in the AH. Moreover, neurokinin-B mRNA level in PH of GDX rats was similar to control. However, excess testosterone levels were effective in significantly inducing the down-regulation of neurokinin-B expression. The basal level of dynorphin mRNA was increased following testosterone treatments in the AH, where we found no significant difference in the level of RFamide-related-peptide mRNA between the experimental groups.

**Conclusion:**

Excess levels of testosterone could act differently from its physiological concentration to regulate hypothalamic androgen sensitive neurons to control GnRH cell.

## Introduction

There are several pathological conditions that are associated with hyper-production of testosterone. Furthermore, androgen abuse among athletes has become a major public health issue (1). It has been reported that supraphysiological levels of androgen have adverse effects on various body systems, such as cardiovascular and central nervous system (2) and perhaps, hypothalamus is mostly affected by testosterone in the brain. 

Gonadotropin-releasing hormone (GnRH) is the hypothalamic neuropeptide that controls the reproductive endocrine system and is considered as ultimate output of the central nervous system driving fertility in all mammals. GnRH induces gonadotropin release from the pituitary gland, leading to production of gonadal sex steroids, which then through positive and negative feedback mechanisms control GnRH neuronal activity. In males, the negative feedback actions of testosterone regulate GnRH, and in turn, gonadotropin (3). GnRH neurons do not express androgen receptors (4); therefore, steroid actions on GnRH neurons must be mediated via upstream steroid-sensitive neurons. 

Discovery of kisspeptin as the most potent stimulator of GnRH neurons (5) has led to better understanding of neuroendocrine reproductive regulation, which is considered as a missing link in the sex steroid feedback control of GnRH. In the rat hypothalamus, kisspeptin cells are located in the anteroventral periventricular (AVPV) and arcuate (ARC) nuclei (6). Although our knowledge about the mechanisms involve in positive feedback action of estradiol are accumulating rapidly, the underlying mechanisms of steroids negative feedback remain unclear. It has been proposed that arcuate kisspeptin cells mediate negative feedback action of sex hormones on GnRH secretion in both male and female rodents; however, pathways through which ARC kisspeptin expressing neurons regulate GnRH neurons are not fully understood (7). 

In addition, several lines of evidence have suggested that ARC kisspeptin neurons regulate pulsatile GnRH secretion via the autosynaptic activity of the neurokinin B (NKB) and dynorphin (DYN), involved in generating the rhythmic discharge of kisspeptin (8, 9). It was reported that NKB by interacting with kisspeptin controls GnRH secretion in the rat (10) and DYN presumably relays inhibitory signals via kisspeptin cells (9). RFamide related peptide-3 (RFRP-3) belongs to the RFamide peptides family and in contrast to kisspeptin, hyperpolarizes GnRH neurons and inhibits the neuropeptide secretion. RFRP-3 is considered as a mammalian ortholog of avian gonadotropin-inhibitory hormone (11-13). 

Since supraphysiological levels of testosterone might act differently from the physiological concentration of the steroid, we hypothesized that GnRH system might also be affected differently after the testosterone intervention. Accordingly, we investigated the impacts of abnormal testosterone levels on GnRH expression in hypothalamus of male rats. Furthermore, we evaluated the expression of two excitatory (kisspeptin and NKB) and two inhibitory (DYN and RFRP-3) neuropeptides upstream of GnRH neurons as possible messengers to relay testosterone signal. 

## Materials and Methods

***Animals***

In the present study, 30 randomly selected adult male Sprague–Dawley rats (weighing 200–250 g) were housed under controlled conditions (12 hr light/dark cycle, 22 ^°^C temperature and ∼40% humidity) and allowed *ad libitum* access to standard food and water. This experiment was approved by Animal Care Committee of the Shahid Beheshti University, Tehran, Iran (approval number: D/920/1010) and carried out in compliance with the recommendations of the Care and Use of Laboratory Animals (National Academy Press, 1996, Washington, USA).

***Gonadectomy and testosterone treatments***

**Figure 1:**
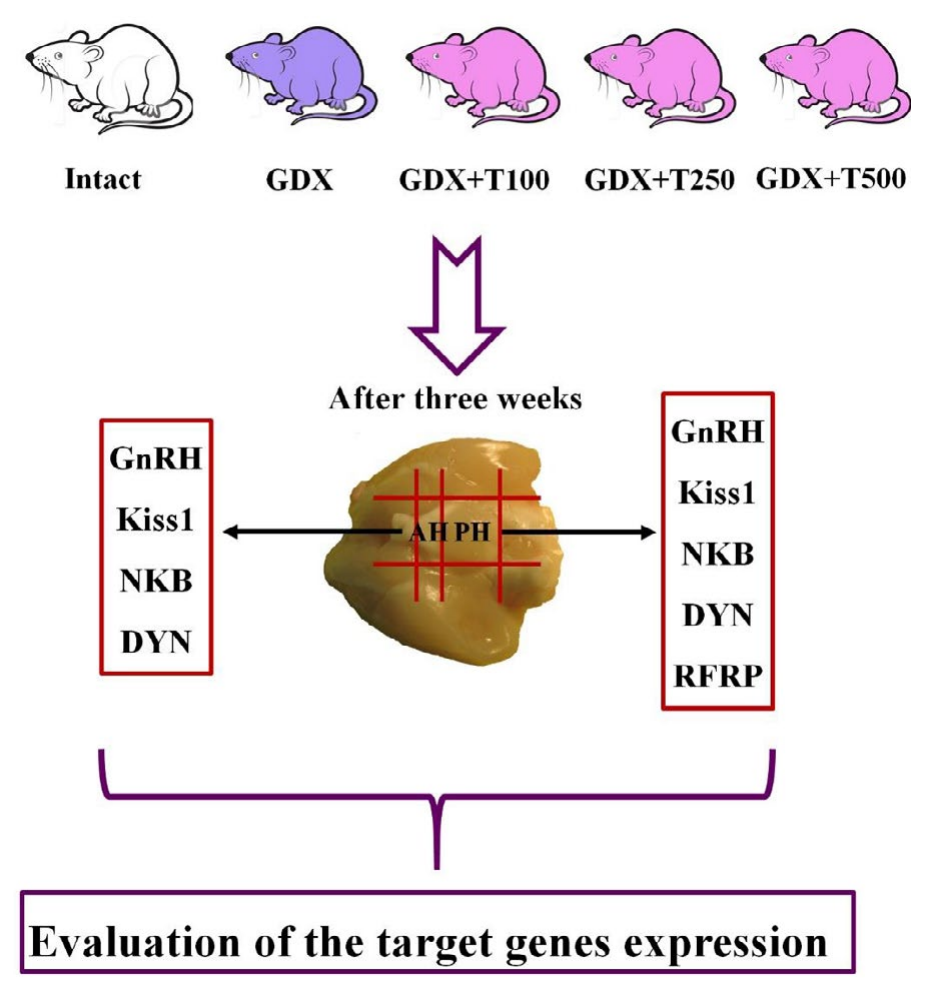
A schema of the experimental design. GDX: gondectomized rats, GDX+T100: gonadectomized rat received 100 mg/kg testosterone undecanoate. GDX+T250: gonadectomized rat received 250 mg/kg testosterone undecanoate. GDX+T500: gonadectomized rat received 500 mg/kg testosterone undecanoate. PH: posterior hypothalamus, AH: anterior hypothalamus

All adult male rats were anesthetized with ketamine (100 mg/kg)/xylazine (10 mg/kg) and divided into five groups: one group of sham-operated non-gonadectomized (CTL group), one group of bilaterally gonadectomized rats received normal saline (GDX group) and three groups of GDX rats received single intramuscular injection of 100 (GDX+T100), 250 (GDX+T250) and 500 (GDX+T500) mg/kg testosterone undecanoate (Nebido, Bayer Pharma, Berlin, Germany) to produce steady state supraphysiological levels of steroid hormone during the experiment. Testosterone undecanoate is a depot preparation allowing continuous slow release of free testosterone from the depot, which reaches the brain. These doses were chosen based on Callies *et al.* work who showed that a single injection of testosterone undecanoate is a well suited for long-term substitution in hypogonadism and is a convenient and effective tool for inducing a desire testosterone level for at least four weeks (14). A schema of experimental design is illustrated in Figure 1.

***Tissue collection***

Three weeks following interventions, rats were anesthetized, brains were immediately removed and hypothalamus was dissected rostral to the anterior border of the optic chiasm, caudal to the posterior border of the mammillary body, 2 mm lateral to the third ventricle and dorsal to the ventral border of the thalamus. Since kisspeptin cells are located in two distinct hypothalamic nuclei, the third coronal cut was made just rostral to the infundibulum in the middle of the optic tract (15). Finally, posterior (PH) and anterior (AH) hypothalami were snap-freezed and stored at -80 ^°^C until further processing. 

***RNA extraction, cDNA synthesis and qRT-PCR***

Total RNAs extraction (YTzol Pure RNA buffer, Yekta Tajhiz Azma, Iran), DNaseI treatment (Thermo Scientific, USA) and cDNA synthesis (Easy cDNA Synthesis kit, Pars Tous, Iran) were performed on the AH and PH samples based on the manufacturer’s instructions.

To evaluate GnRH, Kiss1, NKB, DYN and RFRP-3 transcripts, triplicate reactions were carried out on the cDNA samples. In brief, twenty-microliter reaction mixture was prepared, consisting of RealQ Plus Master Mix Green (Ampliqon, Denmark), first strand cDNA template and appropriate qRT-PCR primer set (specific primers presented in Table 1). Thermo-cycling conditions were 95 ^°^C for 10 min before 40 cycles of 95 ^°^C for 30 sec, 56 ^°^C for 30 sec and 72 ^°^C for 30 sec on an ABI StepOne Real-Time PCR system (Applied Biosystems, USA). Melting curve analysis showed a single amplification peak for each reaction. The Ct value for each target was normalized to the copy number of the beta-actin transcript for each sample. Furthermore, a single band of the expected size was observed for amplified products (5 μl) that were subjected to electrophoresis on a 1% agarose gel stained with ethidium bromide. Fold changes in expression were calculated using the arithmetic formula 2-ΔΔCT (16).

Relative expression of GnRH, Kiss1, NKB and DYN genes were assessed on both the PH and AH samples, while expression of RFRP-3 was evaluated only on the PH.

***Hormone assay***

Serum testosterone and luteinizing hormone (LH) concentrations were measured just before tissue collection, using testosterone [^125^I] RIA kit and rLH [^125^I] RIA kit (Izotop, Hungary) according to the manufacturer’s instructions.

***Statistical analysis***

Obtained data were subjected to the normality test, and comparisons between groups were made by one-way ANOVA plus the Tukey *post hoc* test using GraphPad Prism (Version 7.03, GraphPad Software, Inc., San Diego, CA). The mean±SEM are reported in the text and *P*<0.05 was considered to be statistically significant.

## Results

Three weeks following interventions, serum testosterone concentration was measured to confirm the effectiveness of the drug in producing the desired hormone levels. As shown in Table 2, gonadectomy reduced mean testosterone concentration and androgen replacements, which dose dependently elevated the levels of steroid hormone. In addition, to determine whether different levels of testosterone have different ability to exert negative feedback action on gonadotropin secretion, serum LH concentration was also assessed. Although mean LH concentration was increased by more than 15-fold following gonadectomy, all three androgen treatments preserved gonadotropin secretion as much as the control (Table 2). 

***GnRH expression in response to supraphysiological levels of testosterone***

**Figure 2:**
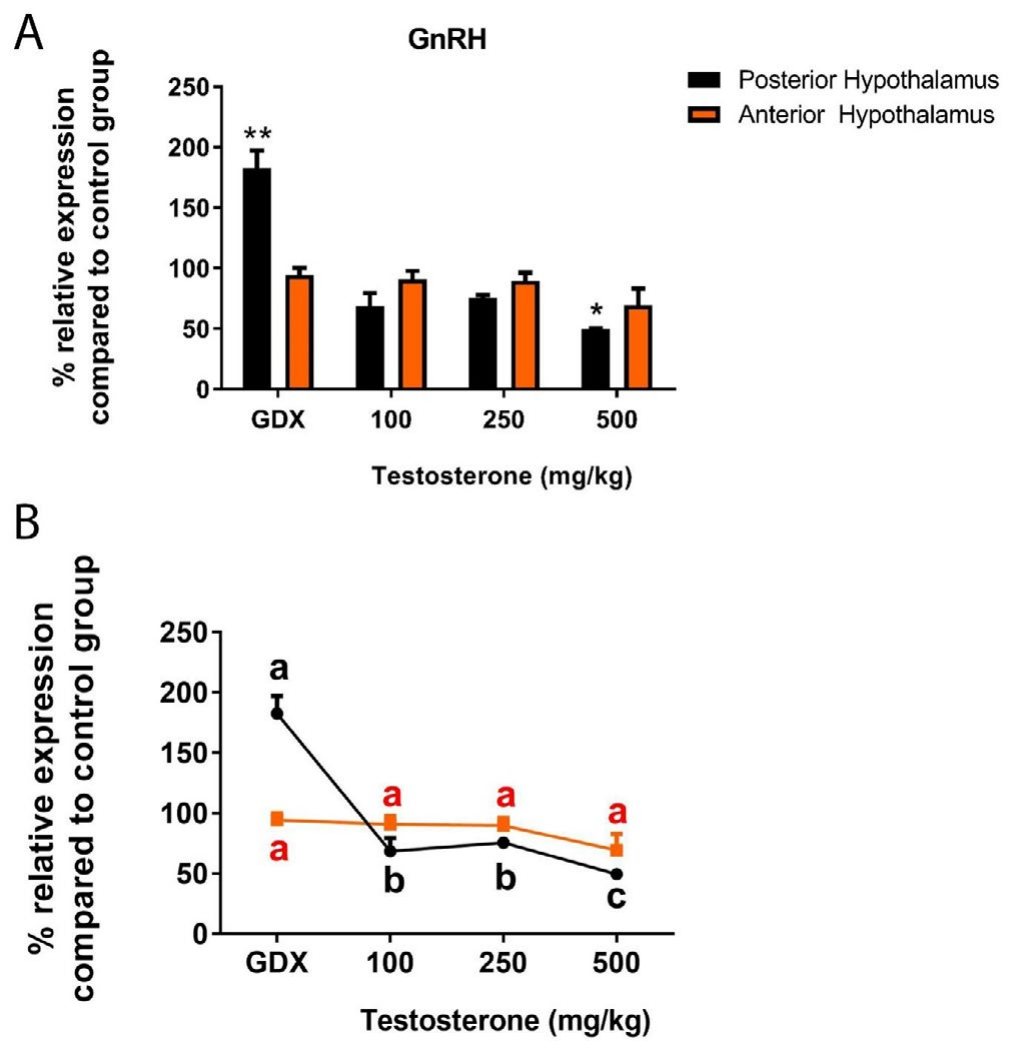
A) Relative expression of gonadotropin-releasing hormone (GnRH) mRNA in the rat posterior and anterior hypothalamus following testosterone interventions compared to control group. *P<0.05, **P<0.01, ns: non-significant; B) Statistical comparison between groups; values with different superscripts are significantly different (*P*<0.05). Data are represented as mean±SEM

Here, quantitative RT–PCR was used to evaluate the ability of gonadectomy and supraphysiological levels of testosterone replacement to regulate gene expression in the AH and PH three weeks after interventions. As shown in Figure 2, GnRH transcript in the PH was high in GDX rats (*P*<0.01), and 500 mg/kg testosterone reduced the level of GnRH mRNA by about 50% (*P*<0.05). GnRH expression in the PH of rats was unaffected by the GDX+T100 or GDX+T250 treatments (*P*>0.05). Neither gonadectomy nor testosterone treatments affected the GnRH transcript in the AH (*P*>0.05).

**Figure 3:**
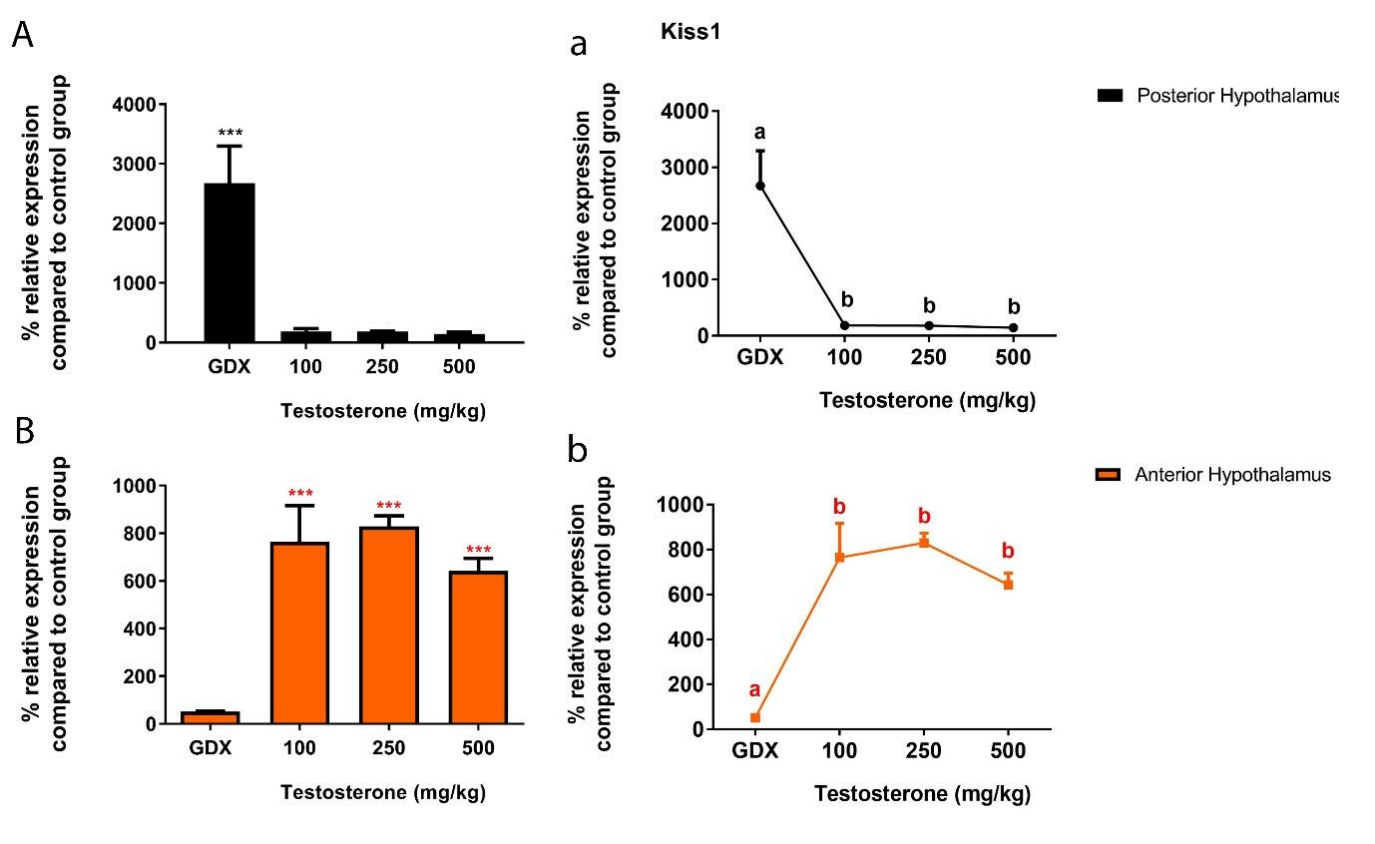
A and B) Relative expression of Kiss1 mRNA in the rat posterior and anterior hypothalamus following testosterone interventions compared to control group. ***P<0.001. a and b) Statistical comparison between groups in the posterior and anterior hypothalamus; values with different superscripts are significantly different (*P*<0.05). Data are represented as mean±SEM

**Figure 4:**
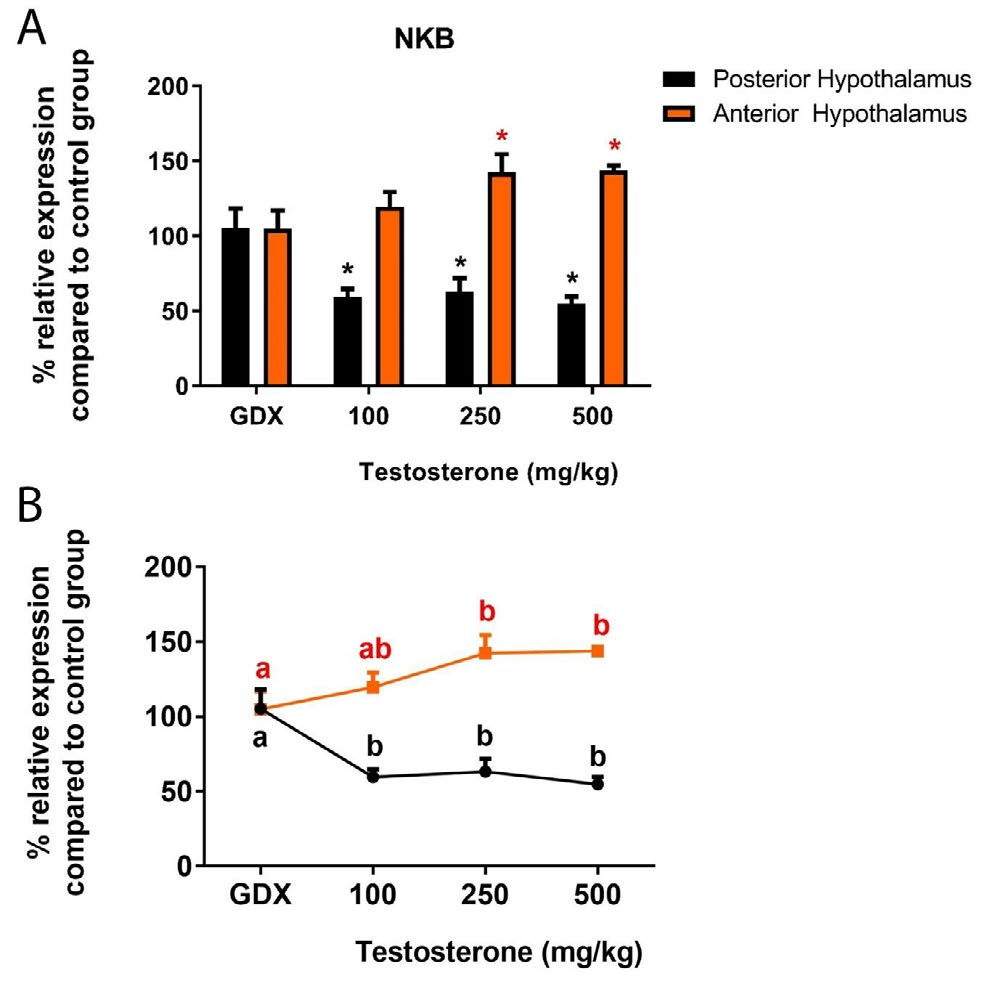
A) Relative expression of neurokinin-B mRNA in the rat posterior and anterior hypothalamus following testosterone interventions compared to control group. *P<0.05. B) Statistical comparison between groups; values with different superscripts are significantly different (*P*<0.05). Data are represented as mean±SEM

**Figure 5:**
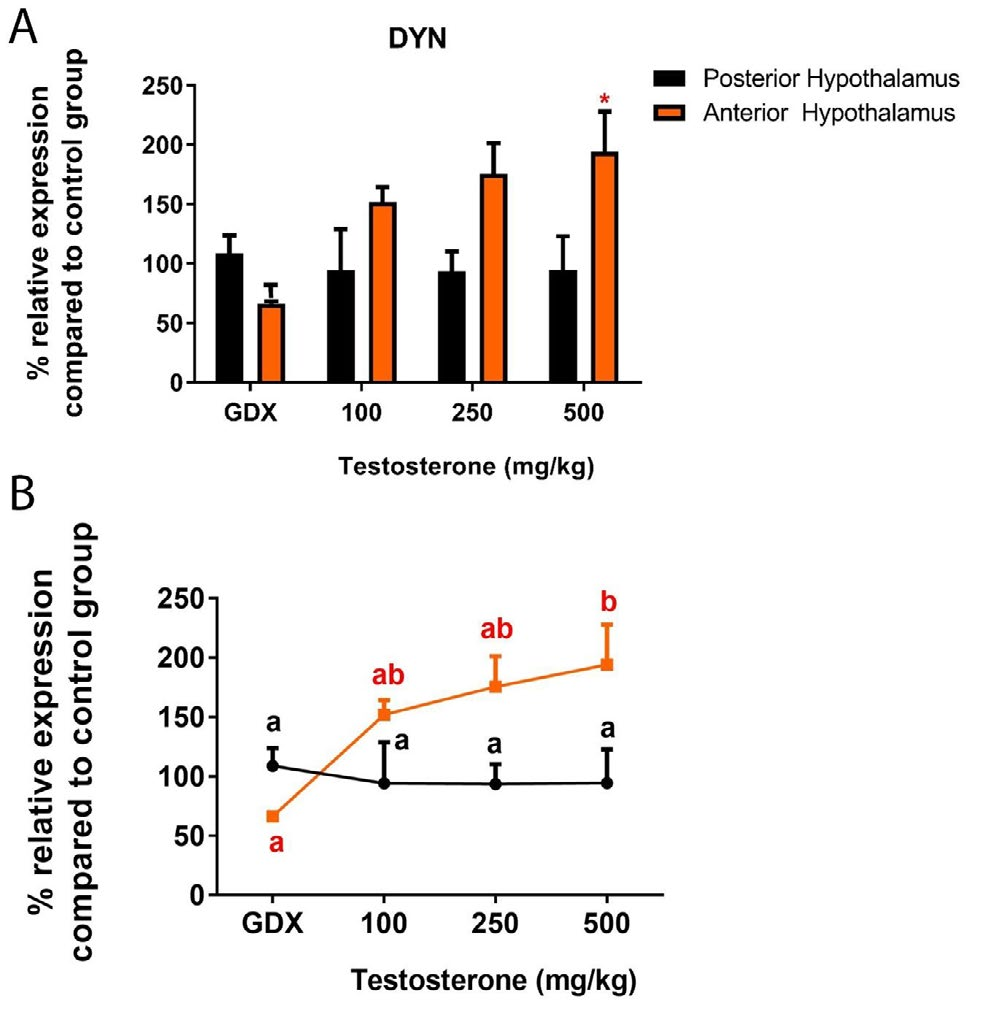
A) Relative expression of dynorphin mRNA in the rat posterior hypothalamus following testosterone interventions compared to control group. *P<0.05. B) Statistical comparison between groups; values with different superscripts are significantly different (*P*<0.05). Data are represented as mean±SEM

**Figure 6:**
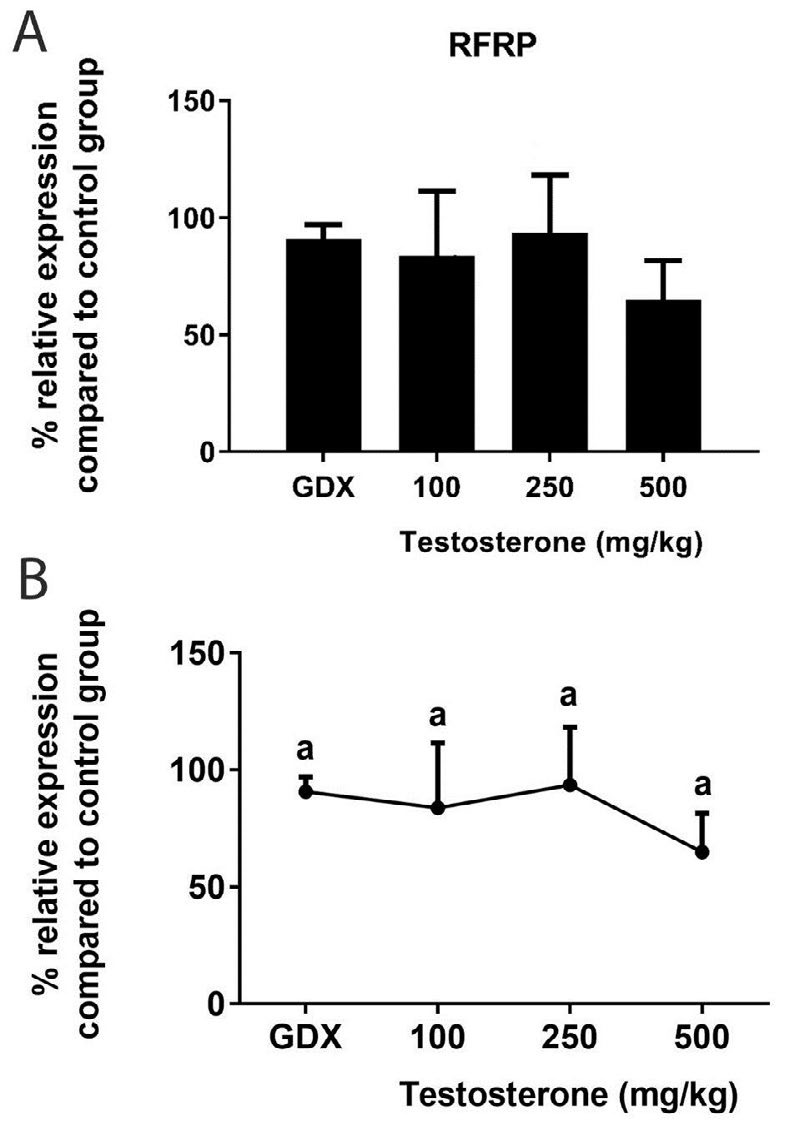
A) Relative expression of RFamide-related-peptide-3 mRNA in the rat posterior hypothalamus following testosterone interventions compared to control group. B) Statistical comparison between groups; values with different superscripts are significantly different (*P*<0.05). Data are represented as mean±SEM

**Table 1:** Primer sequences (5`–3`) used in quantitative polymerase chain reaction (qPCR)

Gene	Accession number	Sequence	Amplicon (bp)
GnRH	NM_012767	F-GCCGCTGTTGTTCTGTTGACTG	133
		R-CCTCCTCCTTGCCCATCTCTTG	
Kiss1	NM_181692	F-TGATCTCGCTGGCTTCTTGGC	98
		R-GGGTTCAGGGTTCACCACAGG	
RFRP-3	NM_023952	F-GAGTCCTGGTCAAGAGCAAC	93
		R-ACTGGCTGGAGGTTTCCTAT	
NKB	NM_019162	F-GGAAGGATTGCTGAAAGTGCTGAG	130
		R-GGGAGTGTCTGGTTGGCTGTTC	
DYN	NM_019374	F-CGGCGTCAGTTCAAGGTGGTG	150
		R-AGCAAGCGAAGGAATAAGCAGAGG	
Actin, Beta	NM_031144	F-TCTATCCTGGCCTCACTGTC	122
		R-AACGCAGCTCAGTAACAGTCC	

GnRH: gonadotropin-releasing hormone, RFRP-3: RFamide related peptide-3, NKB: neurokinin B, DYN: dynorphin.

**Table 2:** Mean ± SEM serum testosterone and luteinizing hormone (LH) concentration three weeks following interventions

	Experimental groups				
	CTL	GDX	GDX+T100	GDX+T250	GDX+T500
Mean testosterone concentration (ng/ml)	1.52±0.21	0.17±0.01	4.41±0.85	8.86±0.63	13.15±2.45
Mean LH concentration (ng/ml)	1.96±0.24	31.94±6.09	1.70±0.21	1.42±0.19	1.83±0.07

CTL: sham-operated non-gonadectomized, GDX: gondectomized rats, GDX+T100: GDX rat received 100 mg/kg testosterone undecanoate. GDX+T250: GDX rat received 250 mg/kg testosterone undecanoate. GDX+T500: GDX rat received 500 mg/kg testosterone undecanoate.

***Effects of testosterone interventions on the kisspeptin and neurokinin-B transcripts***

Next, we evaluated the effects of testosterone on two excitatory neuropeptides upstream of GnRH neurons. It was previously reported that kisspeptin in the ARC and AVPV is regulated by physiological level of testosterone (17). In the present work, we studied the effect of supraphysiological level of testosterone. We found, extremely high (more than 2600%) level of Kiss1 transcript in the PH of GDX rats (*P*<0.001). However, in GDX rats treated with different levels of testosterone, Kiss1 expression was not significantly different than the control rats (*P*>0.05) (Figure 3A). We also found that testosterone replacement elevated Kiss1 transcript level in the AH of GDX animals by around 700% when compared to the control group (*P*<0.001) (Figure 3B). 

NKB is another neuropeptide in the hypothalamus that has crucial function in regulating GnRH system. NKB mRNA in PH of GDX rats was similar to those of the control (*P*>0.05). However, all three different treatments were able to induce a significant down-regulation in gene expression (*P*<0.05) (Figure 4). On the other hand, the level of NKB mRNA in the AH of gonadectomized rats was not significantly different from that of AH in control rats or that of GDX rats receiving 100 mg/kg testosterone (*P*>0.05). However, treatment with 250 or 500 mg/kg testosterone increased NKB transcript slightly but significantly in the AH (*P*<0.05) (Figure 4).

***Dynorphin and RFRP expression in response to testosterone replacements***

Next, the effects of supraphysiological levels of testosterone on two inhibitory neuropeptides – DYN and RFRP– that can regulate GnRH cells, were investigated. DYN expression was not significantly different with/without testosterone in the PH (*P*>0.05) (Figure 5). Furthermore, the basal level of DYN transcript elevated after testosterone interventions in the AH. It should be noted that testosterone only at its highest dose could statistically up-regulate DYN expression (*P*<0.05) (Figure 5). RFRP mRNA in the hypothalamus of GDX rats with and without testosterone was also determined by qRT-PCR. We found no significant difference in the level of RFRP mRNA between the experimental groups (*P*>0.05) (Figure 6). 

## Discussion

In the present study, we confirmed the hypothesis that supraphysiological levels of testosterone can act differently from its physiological concentration to regulate hypothalamic androgen sensitive neurons in order to control GnRH cell. It has been known for while that testosterone exerts negative feedback action on gonadotropin secretion, demonstrated by an elevated release of LH following castration. Although testicular steroid can act at pituitary gonadotrope and/or hypothalamus levels, obtained data from hypothalamo-pituitary disconnection models revealed that the predominant site at which testosterone act to regulate the secretion of LH is within the central nervous system (3). At odds with obvious LH response to testosterone, the effect of testicular steroid on GnRH level were often time inconclusive and contradictory. Previous studies showed either a decrease (18, 19), increase (20, 21) or no change (22, 23) in the content of hypothalamic GnRH following castration. Therefore, we first evaluated whether gonadectomy and testosterone treatments can alter hypothalamic GnRH expression. Our findings showed that gonadectomy increased GnRH mRNA in the PH, and the highest dose of testosterone treatment had a reverse effect on GnRH expression; however, neither gonadectomy nor testosterone treatments affected GnRH mRNA in AH.

There is no doubt that gonadal steroids exert their impacts on GnRH through upstream steroid-sensitive neurons. Kisspeptin neurons as the most important stimulatory input to GnRH cells have been suggested to have a role in testosterone negative feedback mechanism. In this regard, it was reported that castration caused a significant increase in Kiss1 expression in the arcuate of many species such as rats (24), mice (17), goat (25) and monkey (26), which was reversed with testosterone replacement. Similarly, in the present study we observed significant elevation of Kiss1 mRNA in the PH (where ARC nucleus is located) following gonadectomy, while all supraphysiological levels of testosterone decreased GnRH expression to levels found in the control rats. Although kisspeptin cells in ARC are ideally located to act as the missing link in the sex steroid feedback control of GnRH, presently there is little evidence for anatomical connection between GnRH neurons and ARC kisspeptin neurons (7). 

On the other hand, our results showed that excess testosterone exposure can lead to up-regulation of kisspeptin expression (more than 700%) in the AH where AVPV nucleus is located. Also, Kiss1 mRNA in the GDX rats decreased by around 50%, but it was not statistically significant. In female rodents, it is well-documented that AVPV kisspeptin neurons mediate estrogen positive feedback effect to stimulate the preovulatory GnRH/LH surge. In males, kisspeptin cells in AVPV have the same response similar to the female AVPV, but its physiological role is not well-defined as males normally do not generate sex steroid positive feedback (27). Since testosterone regulation of kisspeptin in the AVPV is mediated via estrogen signaling (17), supraphysiological levels of testosterone, which were employed in the present study, could increase Kiss1 expression in the AH after aromatization to estrogen. 

NKB is another neuropeptide with crucial role in the control of reproductive function and inactivating mutations in the genes encoding NKB or its receptors, which results in hypogonadotropic hypogonadism (28). In the rat hypothalamus, majority of NKB mRNA expressing neurons locate in the lateral hypothalamic area (LHA) and ARC (29). Since arcuate NKB neurons co-express androgen receptor (30) and these neurons directly interact with GnRH cells, especially at the median eminence (31), we evaluated the effects of testicular steroid hormone on NKB expression. Here, gonadectomy did not affect NKB expression in the hypothalamus, although, supraphysiological doses of testosterone clearly reduced NKB mRNA in the PH. Previous reports showed either increase (32) or no change (33) in the level of arcuate NKB expression following castration. 

Also in female rats, NKB expression in the ARC was reduced during the afternoon of pro-estrus when circulating estradiol is in its maximum level. Similarly, ovariectomized rats treated with estradiol had fewer NKB mRNAs in the ARC compare to non-treated group (10). Furthermore, based on a postmortem investigation, older men with lower testosterone concentrations had greater numbers of NKB neurons in the infundibular nucleus than young men (34).

On the other hand, we found that in GDX rats receiving 250 or 500 mg/kg testosterone, NKB mRNAs in the AH were slightly but significantly higher than the other groups. Interestingly, estradiol treatment induced NKB expression in the LHA of ovariectomized rats (35). Therefore, based on our findings, high testosterone concentrations might exert dual opposite effects on NKB expression in the ARC and LHA. However, further clarification is required to define whether the effects of testosterone on NKB neurons is direct or mediated via estrogen signaling.

DYN is another element, known for a long time as an inhibitor of gonadotropin secretion (36, 37), which belongs to endogenous opioid peptides family and is considered to mediate the negative feedback effects of progesterone on GnRH pulse frequency (38). Also, a number of pharmacological evidences have proposed that endogenous opioid peptides might be involved in mediating the negative feedback effects of testosterone (3). Our results showed that DYN expression was not affected with or without testosterone in the PH; however, androgen treatment could elevate DYN mRNA in the AH. In line with our findings, Dudek *et al.* found that orchidectomy did not significantly change the number of DYN cells in the ARC of rats (33). 

There are fewer DYN neurons in the infundibular nucleus of the postmenopausal women compared to the premenopausal group (39). Furthermore, ovariectomy reduces the number of DYN mRNA expressing neurons in the preoptic area, AH and ARC in ewes (40), which raise the possibility of inducing DYN expression by estradiol. However, it was reported that expression of DYN is inhibited by estradiol in female mice (8, 41). As DYN mRNAs have been widely distributed throughout the hypothalamus (42); hence, *in situ* analysis such as immunohistochemical study is required to understand the exact localization of the observed changes.

In rodents, RFRP neurons are exclusively located in the dorsomedial hypothalamic nucleus and inhibit GnRH neural activity by sending projections to these cells (11-13). Although androgen receptor was detected in RFRP neurons of male hamster (43), virtually no RFRP neurons in male and female mice co-expressed androgen receptor (44). In addition, estrogen receptor-α mRNA was expressed in approximately 40% of RFRP neurons in hamster (43), 18% in ovariectomized mice (45) and 25% in male and female intact mice (44). Accordingly, we hypothesized that excess testosterone levels might affect RFRP expression directly or following aromatization. Our findings showed that neither gonadectomy nor testosterone treatments affected RFRP mRNA in the hypothalamus. Similarly, there was no statistical difference in RFRP expression between castrated and sham-operated hamsters. Also, the RFRP expression in hamsters treated with testosterone for 4 weeks was not statistically different from castrated group (46). In addition, it was reported that RFRP expression is moderately regulated by estradiol in male and female mice and unaffected by dihydrotestosterone. Therefore, androgen-signaling seems not to be an important regulator of RFRP neurons.

## Limitation

In the present study, we assessed target genes in the AH and PH; however, for more accurate evaluation, dissecting each hypothalamic nucleus is recommended. In addition, for better understating of supraphysiological levels of testosterone effects, evaluation of target genes expression at the level of protein using techniques such as immunohistochemistry or western-blot are recommended. 

## Conclusion

Our results showed that supraphysiological levels of testosterone elevate relative expression of kisspeptin, NKB and DYN in the AH of male rats while decreasing GnRH and NKB mRNAs in the PH. Thus, excess level of the testicular steroid hormone affects neural networks upstream of GnRH neurons differently from its physiological concentration. Therefore, testosterone abuse might have long-term undesirable effects on the brain due to its adverse impacts on various neuropeptides system. 
